# Anthropometric Characteristics of Hospitalised Elderly Women: A Case-Control Study

**DOI:** 10.3390/ijerph8072584

**Published:** 2011-06-24

**Authors:** Slimane Belbraouet, Nearkasen Chau, Ambroise Tebi, Gérard Debry

**Affiliations:** 1 Ecole de Nutrition, 51 A Maillet, Université de Moncton, Moncton, NB, E1A 3E9, Canada; 2 INSERM, U669, IFR25-IFRH, Maison de Solenn, 97 Boulevard de Port Royal, F-75679 Paris cedex 14, France; E-Mail: nearkasen.chau@wanadoo.fr; 3 Université Paris Descartes, UMR-S0669, rue de l’Ecole de Médecine, 75270 Paris, France; 4 Centre de Nutrition Humaine, Université Henri Poincaré-Nancy 1, rue Lionnois, F-54000 Nancy, France; E-Mails: ambroise.tebi@yahoo.fr (A.T.); gerard.debry@yahoo.fr (G.D.)

**Keywords:** anthropometric indicators, malnutrition, diseases, elderly, women

## Abstract

This study assessed the anthropometric status of 451 hospitalised female patients aged 70 or over, at their admission to hospital, in reference to 77 healthy women of the same age. The most frequent diseases were circulatory diseases (40.8%), mental disorders (29.9%), respiratory diseases (12.4%), endocrine and metabolic diseases (11.5%), osteomuscular diseases (8.4%), and traumatisms (6.9%). The differences were significantly high for mid-arm circumference (MAC), triceps skinfold thickness (TSF), weight, weight/height, and body mass index (BMI). The patients with cancers, blood diseases, mental disorders, respiratory disease, digestive diseases, or traumatisms had the lowest values. All the indicators correlated in a similarly negative way with age. The decreased TSF was more pronounced among subjects with respiratory diseases. Measurement of anthropometric indicators, TSF in particular, should be part of preventive measures aimed at reducing malnutrition and its consequences in a hospital setting.

## 1. Introduction

Interrelationships between nutritional status and underlying medical conditions are complex, especially in the elderly [1]. With advancing age, there is a higher incidence of chronic diseases and many bear a nutritional component, either as likely cause or as a result of that condition [2].

Anthropometric measurements provide an indirect evaluation of body composition [3] in individuals and populations with acute and chronic diseases [4]. Various methods of screening for malnutrition may be used, but all have limitations [5]. In addition, the normal ranges have originally been established from healthy individuals and may not appropriate to all ages groups [6]. While the body mass index (BMI) is a widely used parameter [7], little is known about its value in detecting malnutrition in various clinical conditions where changes in body composition may vary according to the nature of pathology. Triceps skinfold thickness (TSF) and mid-arm circumference (MAC) are also commonly used to diagnose malnutrition and, as suggested by Bruun *et al.* [8], these parameters may be appropriate for nutritional screening of the patient population when they are compared to reference data from a healthy population of the same age and area. Consequently, it should be important to determine under what condition the BMI can be either used as a simple tool or associated with other anthropometric parameters to identify the level of malnutrition in the elderly when they are hospitalised.

The aim of this study was to describe the differences of a number of anthropometric indicators (weight, weight/height, BMI, TSF, MAC, arm muscle circumference (AMC), arm muscle area (AMA)) between diseased elderly women with various diseases and healthy elderly women of similar age (70 years or over), both groups from the north-eastern region of France.

## 2. Materials and Methods

The case-control study included a sample of 77 women healthy volunteers randomly chosen among subjects aged 70 or over, living autonomously in residential flats for seniors which serve the same geographical area (north-eastern France), having good health status as assessed by a medical examination (blood pressure, electrocardiogram, pulmonary radiography), and no chronic diseases at the time. These controls were compared to whole 451 women aged 70 or older, and admitted over a 1-year period to the Geriatric Department of Nancy University Hospital. This geriatric unit serves patients from throughout Lorraine (a region in north-eastern France with 2.3 million inhabitants). The study protocol, conducted by one physician on the day after the admission to the geriatric unit, included a questionnaire on socio-demographic characteristics and a medical examination where the main diagnosis and up to five secondary diagnoses were noted. The main diagnosis and up to five secondary diagnoses were coded according to the WHO classification [9]. This study was approved by the Ethics committee of Nancy University Hospital and informed consent was obtained from each subject.

Three measurements were done and then averaged for body height, weight, MAC, and TSF for the left arm. The body height was measured with a Stanley Ltd “microtoise”, MAC with a flexible plastic tape, weight with an Teraillon electronic balance (accuracy of 200 grams), and TSF with a Harpenden skinfold calliper. AMC, AMA, and BMI were defined as follows:

AMC (cm)=MAC (cm)×π×TSF (cm),

[10]

$$\text{AMA }({\text{cm}}^{2})={\text{AMC}}^{2}\;({\text{cm}}^{2})/4\;\pi \times 6.5,$$

[11]

$$\text{BMI }(\text{kg}/{\text{m}}^{2})=\text{weight }(\text{kg})/{\text{height}}^{2}({\text{m}}^{2}).$$

[12]

The comparison of each group’s indexes was done by means of a variance analysis on one factor. The χ^2^ independence test was used to compare the distributions of the subjects' age for both groups. The relationship between two quantitative variables was defined by a Pearson’s correlation coefficient. The covariance analysis, by controlling for age, was done in order to compare the indexes of diseased and healthy women. To analyse the deficiency for each index, the 5th percentile among the healthy women was used as threshold level. The χ^2^ independence test or the Fisher’s exact probability test was performed to compare the deficiency, for an index, in patients suffering from a category of diseases with other patients. The analyses were performed using the Stata program (Stata Corporation, 2007).

## 3. Results

In the diseased subjects, the most frequent pathologies diagnosed were circulatory diseases (40.8%), mental disorders (29.9%), respiratory diseases (12.4%), endocrine and metabolic diseases (11.5%), osteo-muscular diseases (8.4%), and traumatisms (6.9%). The risk patterns of decreased values differed more between anthropometric indicators than between categories of diseases. The diseased subjects had analogous age, height, AMC, and AMA, as well as significantly lower MAC, weight, weight/height, BMI, and TSF (Table 1) than the healthy people. The values of patients with various categories of diseases were lower than those of the healthy people, except for the subjects suffering from infectious or genitourinary diseases. The lowest values were observed in patients with cancers, blood diseases, mental disorders, respiratory illnesses, digestive diseases, or traumatisms. These differences were significant only for MAC, TSF, weight, weight/height, and BMI. In the healthy people, only TSF significantly correlated with age (r = −0.24). In the diseased subjects, age was correlated with all anthropometric indicators; the correlation coefficient was −0.26 for MAC, −0.24 for TSF, −0.23 for height, about −0.20 for weight, AMC, AMA, weight/height, and −0.16 for BMI. Significantly lower values (*P* < 0.01) were found for the subjects aged 85 or over only when compared with those aged 70–74: mean (SD) 51.6 (10.7) *vs.* 57.7 (11.5) kg for weight, 23.5 (3.3) *vs.* 26.2 (3.2) cm for MAC, 1.30 (0.53) *vs.* 1.67 (0.63) cm for TSF, 19.4 (2.3) *vs.* 20.9 (2.2) cm for AMC, 24.0 (7.4) *vs.* 28.8 (7.5) cm^2^ for AMA, 33.9 (6.4) *vs.* 37.5 (6.8) kg/m for weight/height, and 22.7 (4.1) *vs.* 24.4 (4.2) kg/m^2^ for BMI.

Table 2 shows that the decreased values (below the 5^th^ percentile values in the healthy people) were common and greatly differed between anthropometric indicators: 35.1% for TSF, 33.7% for MAC, about 28% for weight and BMI, 23.8% for weight/height, and 17.5% for AMA. The frequency of deficiency increased with age and was much higher among the diseased subjects aged 85 or over: 51.6% for TSF, 46.0% for MAC, about 33% for weight and BMI, 30.5% for weight/height, and 17.5% for AMA. The percentage of deficiency did not differ among the diseased subjects except for those with respiratory diseases of whom 48.2% (35.1% for all diseased people as a whole) showed a deficiency for TSF.

## 4. Discussion

The present study shows the risk pattern of decreased values for the anthropometric status among hospitalised elderly women. The hospitalised elderly women and the healthy women were of the same age and hailed from the same region. The distribution of diseases among patients was similar to the one among patients seen on an annual basis by general practitioners in Lorraine [13].

The cut-off values for the anthropometric indicators were defined by the 5th percentile in the healthy women involved in the study. Except for TSF, all anthropometric parameters were similar to those found by Burr and Philipps [14], Falciglia *et al.* [15], and Kubena *et al.* [16]. Concerning mean values, although the healthy women had lower MAC and AMC, they showed higher TSF than the normative values proposed by Jelliffe [10] for adults. They had similar MAC, AMC, TSF, and BMI to those reported by other studies [15–18]. Our values were close for AMC and AMA but higher for MAC, TSF, and BMI than those from Woo *et al.* [19] and Volkert *et al*. [20]. Frisancho [12] found, among the American elderly, similar values for BMI and TSF but higher values for MAC and AMA.

Deficiency was common for most disease categories. The diseased women at the moment of admission to a hospital showed lower values of all the parameters than the subjects belonging to the healthy women group, except for AMC and AMA. Our study reveals that TSF was the most decreased anthropometric parameter. The ratio of TSF between the average values of diseased and healthy people equalled 75% *vs.* about 90% for weight, MAC, weight/height, and BMI. Decreased TSF has already been reported [21]. The increase or decrease in body weight is generally paralleled by similar changes in subcutaneous fat [21]. TSF would be a body fat-related index [22] and is considered as a measure of subcutaneous adipose tissue [4]. This was also reported in the French population by Campillo *et al.* [7] and Gillette-Guyonnet *et al.* [23]. BMI is correlated with all of the parameters in both diseased and healthy people. It is well known that BMI is an indicator of fat mass but previous studies have shown that BMI also correlates strongly with lean body mass [7].

In the patients, all of the anthropometric indicators negatively correlated with age, and noticeable decreased values were found in subjects aged 85 or over. Our study found that TSF and MAC were more decreased among the older subjects, especially among those aged 85 or over. This suggests that old age leads to a loss of fat in addition to a loss of muscle as stated by Burr & Phillips [14] and Delarue *et al.* [17]. Most subjects aged 80 or over have at least one impairment and half of them have 3 impairments or more [24]. Nutritional control of these subjects is important as their malnutrition and impairments may result in reduced functional capacity [25]. It is fairly well known that BMI increases with age through the adult years and that a levelling or decline occurs later in life. In fact, age may be the most consistent and predominant factor related to weight variation over the course of a lifetime [26].

In addition, the risk patterns of decreased anthropometric measurements differed between diseases. Patients suffering from blood, nervous system, cardio-vascular, respiratory, digestive, and osteo-muscular diseases, cancers, mental disorders, and traumatisms had markedly lower MAC, TSF, weight, weight/height and BMI. Patients with endocrine and metabolic diseases only had significantly lower TSF. These results are consistent with the relationships between nutrition and chronic disease emphasised by other researchers like Belbraouet *et al.* [27] and Bergström and Lindholm [28]. It is well known that cancers, traumatisms, and cardiovascular diseases are major causes of malnutrition in the elderly [6]. In the present study, bed-ridden patients were excluded because it was not possible to measure their body height as well as their body weight. They represented 11.7%, 11.1%, 18.1%, and 26.9% for the 70–74, 75–79, 80–84, and 85 or over age groups respectively. Our results may thus be underestimated, especially for the older patients. Our findings shed light on the risk patterns of decreased values for a number of anthropometric indicators among patients with most categories of diseases and age groups.

## 5. Conclusions

The present study demonstrates that a great proportion of elderly women suffer from malnutrition at the moment of hospitalisation and that this malnutrition is rather moderate and while it affects mainly the body fat it also affects the muscle among the patients with a wide range of diseases and the people aged 85 or over. A number of pathologies were observed which were strongly associated with malnutrition: blood, nervous system, cardio-vascular, digestive, and osteo-muscular diseases, cancers, mental disorders, traumatisms, endocrine and metabolic diseases, and more particularly respiratory illnesses. TSF appears to be a useful index in evaluating the nutritional status of elderly patients, although BMI and MAC remain also useful. AMC and AMA are important in assessing the high prevalence malnutrition for subjects aged 85 or over. The proportion of people in this age category will probably increase in the general population due to the lengthening of life expectancy. Greater efforts should be made to prevent the development of nutritional deficits during hospitalisation, and physicians and nutritionists should remember that anthropometric parameters are useful for assessing the nutritional status of patients.

## Figures and Tables

**Table 1:** Anthropometric indices of healthy women and of diseased women according to disease category: mean (SD).

	Healthy women	Diseased women													
		Total	Infectious diseases	Cancers	Endocrine metabolic diseases	Blood diseases	Mental disturbance	Nervous system diseases	Cardiovascular diseases	Respiratory diseases	Digestive diseases	Genitourinary diseases	Osteomuscular diseases	Undefined symptom	Traumatisms
No of subjects a	77 (77)	451 (370)	21 (16)	18 (16)	52 (38)	24 (18)	135 (126)	22 (19)	184 (132)	56 (44)	24 (21)	11 (9)	38 (32)	54 (46)	31 (25)
Age (yr)	80.1 (5.1)	81.5 (6.0)	81.2 (6.4)	81.2 (4.4)	81.8 (5.7)	82.8 (6.1)	80.0 (5.5)	79.3 (6.4)	82.1 (6.0)	82.3 (6.4)	81.7 (5.6)	80.8 (5.2)	81.2 (6.1)	80.4 (6.7)	82.6 (5.6)
Height (m)	1.51 (0.06)	1.52 (0.06)	1.52 (0.06)	1.51 (0.03)	1.52 (0.05)	1.51 (0.07)	1.52 (0.06)	1.51 (0.06)	1.52 (0.07)	1.52 (.06)	1.52 (0.07)	1.55 (0.11)	1.50 (0.09)	1.52 (0.07)	1.50 (0.05)
Weight (kg)	62.6 (10)	55.6 ‡ (12.4)	60.1 (14)	52.6 ‡ (12)	57.4 (16)	53.8 † (9)	54.6 ‡ (13)	54.9 * (11)	55.6 * (12)	55.4 ‡ (14)	55.6 † (13)	61.4 (17)	58.7 (17)	56.6 † (13)	52.9 ‡ (11)
MAC (cm)	27.0 (3.0)	25.0 ‡ (3.9)	25.9 (5.3)	24.5 † (4.1)	25.5 (4.4)	24.4 * (3.4)	25.1 * (3.8)	25.6 * (5.1)	24.9 (3.7)	24.4 ‡ (4.3)	24.2 ‡ (3.7)	25.1 (4.4)	25.5 * (4.5)	25.3 ‡ (3.6)	24.7 ‡ (3.8)
TSF (cm)	2.08 (0.61)	1.56 ‡ (0.68)	1.73 (0.78)	1.38 ‡ (0.65)	1.65 * (0.69)	1.52 † (0.71)	1.49 ‡ (0.62)	1.57 ‡ (0.51)	1.60 * (0.70)	1.45 ‡ (.71)	1.33 ‡ (0.58)	1.40 (0.85)	1.57 ‡ (0.62)	1.70 ‡ (0.74)	1.45 ‡ (0.67)
AMC (cm)	20.5 (2.0)	20.1 (2.5)	20.5 (3.6)	20.1 (2.5)	20.3 (2.9)	19.7 (2.0)	20.4 (2.5)	20.7 (2.0)	19.9 (2.4)	19.8 (2.8)	20.0 (2.4)	20.7 (3.0)	20.5 (3.3)	19.9 (2.2)	20.2 (2.4)
AMA (cm^2^)	27.3 (6.4)	26.3 (8.5)	27.7 (13)	26.2 (7.8)	27.1 (10)	24.6 (6.3)	27.1 (8.7)	27.9 (6.5)	25.6 (8.1)	25.4 (9.0)	25.9 (7.7)	28.2 (10)	27.9 (12)	25.4 (6.9)	26.4 (7.8)
W/H (kg/m)	41.3 (6.3)	36.5 ‡ (7.6)	39.6 (9.4)	35.3 ‡ (7.6)	37.7 (9.1)	35.9 † (6.7)	35.6 ‡ (7.3)	36.4 † (7.0)	36.6 * (7.8)	35.9 ‡ (8.7)	36.4 † (8.2)	39.5 (9.5)	38.0 * (8.9)	37.7 † (7.1)	35.1 ‡ (7.6)
BMI (kg/m^2^)	27.4 (4.2)	24.0 ‡ (4.9)	26.0 (6.1)	23.3 ‡ (5.1)	24.8 (5.8)	23.9 † (4.7)	23.4 ‡ (4.6)	24.2 * (4.7)	24.1 * (4.9)	23.6 ‡ (5.5)	23.9 † (5.1)	25.6 (5.9)	25.4 * (5.4)	24.8 † (4.5)	23.5 ‡ (5.0)

* *P* < 0.05

† *P* < 0.01

‡ *P* < 0.001

a In parentheses: number of subjects with measurements of body weight, body height, and BMI; The comparison between the patients with each category of diseases to the healthy people was made using the covariance analysis by taking age into account; MAC: mid-arm circumference, TSF: triceps skinfold thickness, AMC: arm muscle circumference, AMA: arm muscle area, W/H: weight/height, BMI: body mass index; Note: the sum of the numbers of subjects with various diseases was higher than 451 due to poly-pathologies.

**Table 2:** Frequency of diseased subjects (%) with decreased values for various anthropometric indices according to age and disease.

Threshold values b	No. of subjects a	Weight (kg)	MAC (cm)	TSF (cm)	AMA (cm^2^)	W/H (kg/m)	BMI (kg/m^2^)
		*47.7*	*23.1*	*1.21*	*18.6*	*31.0*	*21.3*
**Total**	451 (370)	27.0	33.7	35.1	17.5	23.8	28.6
Age (yr) c							
70–74	60 (53)	16.1	18.6	20.3	6.8	15.1	22.6
75–79	117 (104)	25.4	33.6 *	30.2	13.8	24.0	28.8
80–84	144 (118)	27.7	29.2	30.6	15.3	22.0	26.3
85 or over	130 (95)	33.3 *	46.0‡	51.6‡	28.6‡	30.5 *	34.7
**Disease**^d^							
Infectious diseases	21 (16)	17.6	33.5	35.6	14.3	25.0	18.7
Cancers	18 (16)	41.2	52.9	41.2	17.7	37.5	50.0
Endocrine and metabolic diseases	52 (38)	26.7	27.4	25.5	19.6	23.7	29.0
Blood diseases	24 (18)	31.8	40.9	40.9	31.8	22.2	27.8
Mental disturbance	135 (126)	29.8	30.4	39.3	14.1	25.4	31.7
Nervous system diseases	22 (19)	25.0	22.7	22.7	4.5	21.0	26.3
Cardio-vascular diseases	184 (132)	25.5	35.7	35.2	20.9	22.0	26.5
Respiratory diseases	56 (44)	32.6	38.9	48.2*	18.5	29.5	36.4
Digestive diseases	24 (21)	34.8	50.0	45.8	20.8	33.3	33.3
Genitourinary diseases	11 (9)	22.2	45.4	63.6	18.2	22.2	33.3
Osteo-muscular diseases	38 (32)	30.3	31.6	34.2	15.8	18.7	21.9
Undefined symptom	54 (46)	22.4	28.3	22.6*	15.1	17.4	23.9
Traumatisms	31	33.3	41.9	45.2	16.1	32.0	24.0
**Number of diagnoses**^e^							
1	229 (191)	25.0	32.0	34.0	17.4	21.3	28.0
2	145 (115)	33.1	40.8	40.1	21.1	31.3*	32.2
3 or more	56 (44)	20.4	23.2	26.8	8.9	15.9	22.7

* *P* < 0.05

† *P* < 0.01

‡ *P* < 0.001; MAC: mid-arm circumference, TSF: triceps skinfold thickness, AMC: arm muscle circumference, AMA: arm muscle area, W/H: weight/height, BMI: body mass index

a In parentheses: number of subjects with measurements of body weight, body height, and BMI

b Defined by the 5^th^ percentiles in the healthy subjects

c Each group was compared with the 70–74 age group

d Each category of disease was compared with all the other diseases

e The undefined symptom was excluded. Each group was compared with that with one diagnostic only; Notes: The threshold values used for MAC, TSF, AMC, and BMI were close enough to those from the other authors. The patients with various categories of diseases and the healthy people were of similar age.
